# Human Salivary Protein Histatin 5 Has Potent Bactericidal Activity against ESKAPE Pathogens

**DOI:** 10.3389/fcimb.2017.00041

**Published:** 2017-02-15

**Authors:** Han Du, Sumant Puri, Andrew McCall, Hannah L. Norris, Thomas Russo, Mira Edgerton

**Affiliations:** ^1^Department of Oral Biology, School of Dental Medicine, University at BuffaloBuffalo, NY, USA; ^2^Veterans Administration Western New York Healthcare SystemBuffalo, NY, USA; ^3^The Department of Medicine, University at BuffaloBuffalo, NY, USA; ^4^Department of Microbiology and Immunology, University at BuffaloBuffalo, NY, USA; ^5^The Witebsky Center for Microbial Pathogenesis, University at BuffaloBuffalo, NY, USA

**Keywords:** ESKAPE, antimicrobial peptide, Histatin 5, *Candida albicans*, bactericidal activity

## Abstract

ESKAPE (*Enterococcus faecium*, *Staphylococcus aureus*, *Klebsiella pneumoniae*, *Acinetobacter baumanni*, *Pseudomonas aeruginosa*, and *Enterobacter* species) pathogens have characteristic multiple-drug resistance and cause an increasing number of nosocomial infections worldwide. Peptide-based therapeutics to treat ESKAPE infections might be an alternative to conventional antibiotics. Histatin 5 (Hst 5) is a salivary cationic histidine-rich peptide produced only in humans and higher primates. It has high antifungal activity against *Candida albicans* through an energy-dependent, non-lytic process; but its bactericidal effects are less known. We found Hst 5 has bactericidal activity against *S. aureus* (60–70% killing) and *A. baumannii* (85–90% killing) in 10 and 100 mM sodium phosphate buffer (NaPB), while killing of >99% of *P. aeruginosa*, 60–80% *E. cloacae* and 20–60% of *E. faecium* was found in 10 mM NaPB. Hst 5 killed 60% of biofilm cells of *P. aeruginosa*, but had reduced activity against biofilms of *S. aureus* and *A. baumannii*. Hst 5 killed 20% of *K. pneumonia* biofilm cells but not planktonic cells. Binding and uptake studies using FITC-labeled Hst 5 showed *E. faecium and E. cloacae* killing required Hst 5 internalization and was energy dependent, while bactericidal activity was rapid against *P. aeruginosa* and *A. baumannii* suggesting membrane disruption. Hst 5-mediated killing of *S. aureus* was both non-lytic and energy independent. Additionally, we found that spermidine conjugated Hst 5 (Hst5-Spd) had improved killing activity against *E. faecium, E. cloacae*, and *A. baumannii*. Hst 5 or its derivative has antibacterial activity against five out of six ESKAPE pathogens and may be an alternative treatment for these infections.

## Introduction

Bacteria causing nosocomial infections are increasingly becoming drug resistant, posing a serious health concern, especially in the Intensive Care Units (ICUs) and in surgical wards. A recent report estimated a total of 722,000 of such drug-resistant infections, with an astonishing 75,000 resulting in deaths (Magill et al., 2014). A group of bacterial pathogens including *Enterococcus faecium*, *Staphylococcus aureus*, *Klebsiella pneumoniae*, *Acinetobacter baumanii*, *Pseudomonas aeruginosa*, and *Enterobacter* species (referred to as ESKAPE pathogens) have been of particular concern in this regard (Rice, 2010). Two-thirds of all healthcare-associated infections are ESKAPE related (Boucher et al., 2009), and many of these bacteria use multiple drug resistance mechanisms to “eskape” killing by both conventional and some newer generation antibiotics (Rice, 2008).

Most nosocomial infections result from an exogenous inoculum including the hospital environment and medical personnel, resulting in colonization of ESKAPE pathogens within various patient niches. *E. faecium, K. pneumoniae, P. aeruginosa*, and *Enterobacter* species are common residents of mucosal surfaces such as the oral cavity and the gastrointestinal tract (Keller et al., 1998; Podschun and Ullmann, 1998; Rice, 2010; Vu and Carvalho, 2011). *S. aureus* is a skin commensal and *A. baumanni* has been known to have comparatively long survival rates on epithelial surfaces (e.g., skin; Houang et al., 1998; Coates et al., 2014). Also, *P. aeruginosa* and *K. pneumonia* were detected in 50 and 31%, respectively, of HIV-positive patients' salivas in hospital settings (Lopes et al., 2015). Thus, colonization of the oral cavity with *K. pneumoniae, P. aeruginosa*, and *Enterobacter species* can serve as a potential inoculum source for pneumonia (Sands et al., 2016). Furthermore, *Candida albicans*, an oral commensal fungus also present in the oral cavity, can contribute to a more robust *P. aeruginosa* lung infection if the two are present together (Lindsay and Hogan, 2014).

Salivary innate immunity is the first line of defense against transient and pathobionts in the oral cavity (Salvatori et al., 2016). This is illustrated by the example of salivary Histatin 5 (Hst 5), a cationic protein that has strong fungicidal activity against *C. albicans* (Puri and Edgerton, 2014). Changes in the levels of Hst 5 associated with immunodeficiency may increase susceptibility to oral candidiasis caused by *C. albicans* (Khan et al., 2013). However, Hst 5 was shown to be largely ineffective against oral commensal bacteria as well as cariogenic or periodontal pathogens (Devine and Hancock, 2002; Groenink et al., 2003; Dale and Fredericks, 2005).

However, antimicrobial activity of Hst 5 against the ESKAPE pathogens has never been examined. Hst 5-mediated candidacidal activity is an energy-dependent, non-lytic process, where multiple intracellular targets are affected after Hst 5 has been transported to the cytosol in an energy dependent manner (Puri and Edgerton, 2014). Given the fact that most ESKAPE pathogens are not present in large numbers in the oral cavity of healthy humans, and do not cause any known oral diseases, we hypothesized that salivary Hst 5 may play a substantial role in keeping at least some of the ESKAPE pathogens in check within the oral environment. Here we show for the first time that Hst 5 demonstrates killing activity against all ESKAPE pathogens except *K. pneumoniae*. Killing against some ESKAPE pathogens was lytic in nature; however, as for *C. albicans*, more than one mechanism of killing seems to be involved in Hst 5 activity against ESKAPE bacteria.

## Materials and methods

### Strains, culture conditions, and peptides

All ESKAPE strains used in this study are clinical isolates and are listed in Table 1. These isolates were collected over the past 20 years directly from the clinical microbiology laboratory at the University at Buffalo, which performed strain identification by approved laboratory methodology. Isolates from de-identified plates were frozen and stored at −80°C. *Enterococcus faecium* and *Staphylococcus aureus* were grown in Trypticase soy broth (Sigma-Aldrich; TSB) or TSB agar. All other strains were cultured in Luria-Bertani (LB; BD Biosciences) broth or on LB agar. The incubation temperature for all strains was 37°C. Histatin 5 (Hst 5), FITC-labeled Hst 5 (F-Hst 5), and spermidine conjugated Histatin 5 (Hst 5-Spd) were synthesized by Genemed Synthesis Inc. (San Antonio, Texas). Spermidine was purchased from Sigma-Aldrich.

**Table 1:** **ESKAPE strains used in this study**.

**Species**	**Strain #**	**Gram staining**	Source^*^
*Enterococcus faecium*	94-346-1139	+	Buffalo, NY
*Staphylococcus aureus*	299-0432	+	Buffalo, NY
*Staphylococcus aureus* (MRSA)	94-292-1348	+	Buffalo, NY
*Klebsiella pneumoniae*	Montreal #33	-	Montreal, Canada
*Acinetobacter baumannii*	AB307-0294	-	Buffalo, NY (Adams et al., 2008)
*Acinetobacter baumannii*	HUMC1	-	Luo et al., 2012
*Pseudomonas aeruginosa*	94-323-0635	-	Buffalo, NY
*Pseudomonas aeruginosa*	PAO1	-	Sutton lab, Buffalo, NY
*Enterobacter cloacae*	94-293-0624	-	Buffalo, NY

* *Isolates from the University at Buffalo were collected over the past 20 years directly from the clinical microbiology laboratory, which performed strain identification by approved laboratory methodology*.

### Bactericidal assay

Bactericidal assays were performed by microdilution plate method as described for candidacidal assays (Jang et al., 2010), with some modifications. Briefly, a single colony of each strain was inoculated into 10 mL of media and grown for 16 h at 37°C, cultures were diluted to an OD_600_ = 0.1 in fresh media and incubated with shaking at 37°C to mid-log phase (OD_600_ ≈ 1.0). Cultures were spun down at 1800 × g for 3 min and washed three times with 10 mM sodium phosphate buffer (pH 7.4; NaPB). Cells were re-suspended (10^7^ cells/mL) in 200 μL NaPB (control) or in NaPB containing peptides (30 μM); then incubated at 37°C (except for *S. aureus* that was incubated at room temperature for optimal growth). Aliquots were removed after 1 min, 1 h and 5 h incubation, then were serially diluted with phosphate buffered saline (PBS; 137 mM NaCl, 10 mM sodium phosphate, 2.7 mM KCl, pH of 7.4); plated and incubated for 24 h at 37°C to visualize surviving CFUs. Assays were performed in triplicate. Percentage killing was calculated as [1 − number of colonies from peptide-treated cells/mean number of colonies from control cells] × 100%. Bactericidal assays were also performed in 100 mM NaPB and PBS.

### Live cell imaging of F-Hst 5 and PI uptake by time-lapse confocal microscopy

Confocal microscopy was performed using live cells of six ESKAPE strains attached to chambered borosilicate coverglass (Lab-Tek #155411, 8 chamber). Cells in mid-log phase were collected and washed three times and re-suspended in 10 mM NaPB. Cells (2 × 10^7^ in 200 μL NaPB) were added to each well, allowed to settle for 15 min, then propidium iodide (PI; 2 μg/mL; Sigma) and F-Hst 5 (30 μM) were added. Time-lapse confocal images were recorded immediately after addition of peptide and PI. Confocal images were acquired with a Zeiss LSM510 Meta Confocal Microscope (Carl Zeiss, Germany) using a Plan Apochromat 63X/1.4 Oil Immersion objective. For *E. faecium, S. aureus, K. pneumoniae*, and *E. cloacae*, images were collected every 10 min; and for *A. baumannii* and *P. aeruginosa* every 2 min. In order to detect F-Hst 5 and PI simultaneously, the 488 nm line of the argon ion laser and a 561 nm DPSS laser were directed over an HFT UV/488/561 beam splitter, and fluorescence was detected using a Mirror or NFT 565 beam splitter in combination with a BP 500–550 filter for F-Hst 5 and an LP 575 or BP 650–710 filter for PI detection. ImageJ software was used for image acquisition and analysis.

### Biofilm killing assays

To evaluate killing of bacterial biofilms by Hst 5, cells were inoculated at 1 × 10^7^ cells/ml into 96 well plates and grown overnight at 37°C and 90 RPM for 12 h to form biofilms. After 12 h, spent media was replaced with fresh media, and biofilms were grown for another 4 h in the same conditions. Media was removed and biofilms were incubated with 30 μM Hst 5 suspended in 10 μM NaPB with 10 μM propidium iodide (PI) for 1 h at 37°C, except for *S. aureus* which was incubated at RT. After 1 h, biofilms were gently scraped to remove them from each well, diluted, and placed on microscope slides for image acquisition using a Zeiss Axioobserver.Z1 inverted fluorescent microscope, and an Axiocam 503M camera. Percent killing was calculated as the number of PI positive cells divided by the total number of cells from at least 25 fields from two independent wells.

### Hst 5 killing activity determination in energy deprived cells

Overnight cultures of *E. faecium, S. aureus, A. baumannii*, and *E. cloacae* were diluted and pre-incubated with 10% of media with or without 10 mM NaN_3_ at 37°C. *P. aeruginosa* cells were pre-incubated in 10 mM NaPB with 10 mM NaN_3_ at 37°C due to altered cell viability in the presence of 10% media with NaN_3_. After 3 h incubation, cells were collected by centrifugation and washed two times with NaPB; then cells were used for Hst 5 bactericidal assay in NaPB as described above.

### Susceptibility assay

Minimum inhibitory concentration (MIC) was determined by broth microdilution method based on the guide of Clinical and Laboratory Standards Institute (Clinical Laboratory Standards Institute, 2012) with some modifications. Briefly, all bacterial strains except *K. pneumoniae* were cultured in respective media and grown overnight at 37°C. Cells were washed with NaPB buffer, diluted in 10% of Mueller-Hinton (MH) broth (Sigma) to obtain a concentration of 5 × 10^6^ CFU/mL that were used as the inoculum. Hst 5 peptide was serially diluted in 10% MH broth (since it is inactive in undiluted broth) in 96-well flat-bottomed plates (Falcon). After adding equal volume of target bacterial suspension to the peptide solution, the 96-well plates were incubated at 37°C for 24 h. The MIC values were determined by visibly recording the lowest concentration of Hst 5 that inhibited growth.

### Statistics

Statistical analyses were performed using GraphPad Prism software version 5.0 (GraphPad Software, San Diego, CA, USA) using unpaired Student's *t*-tests. Differences of *P* < 0.05 were considered significant. All experiments were performed at least thrice.

## Results

### Hst 5 has strong bactericidal activity against four ESKAPE pathogens

**Figure 1:**
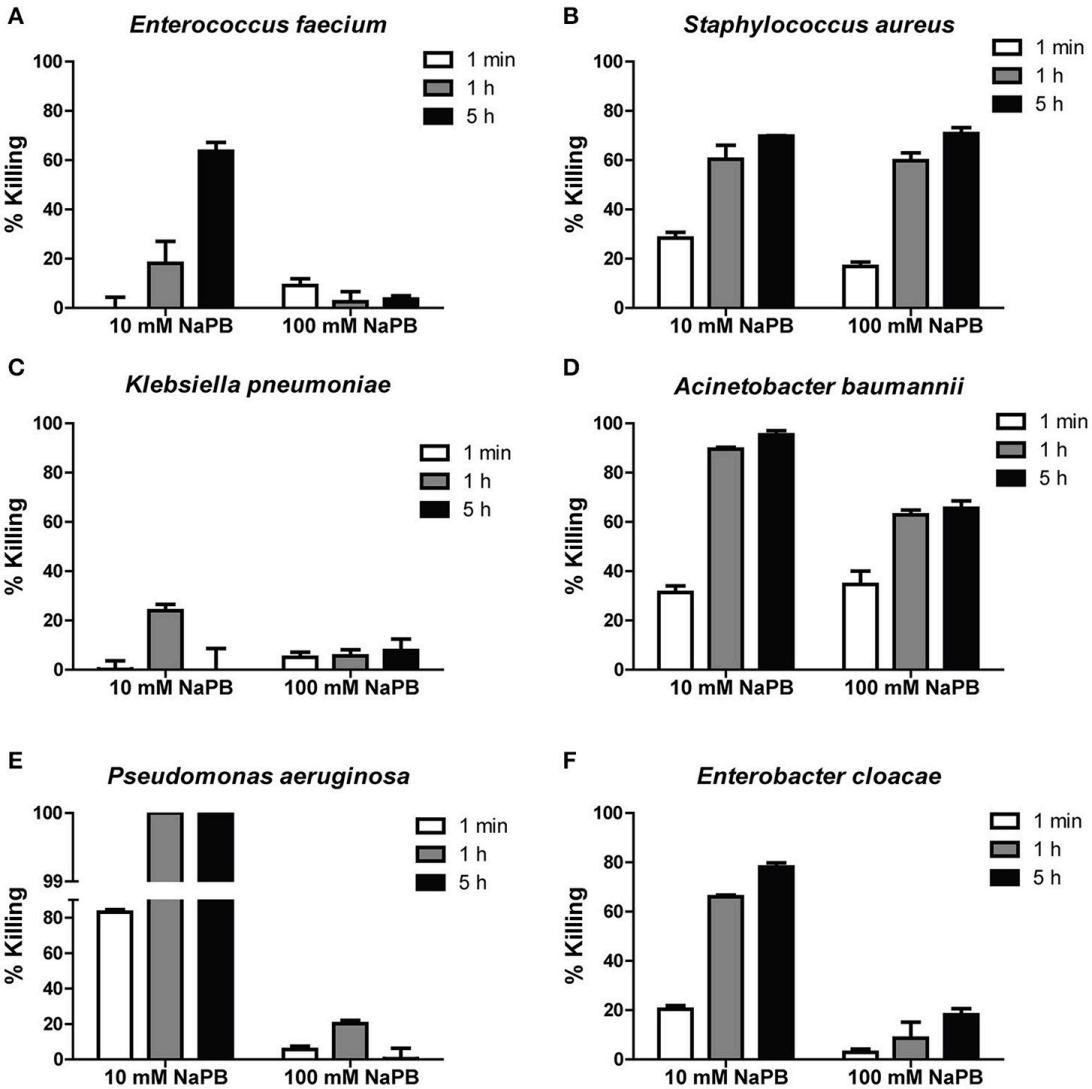
**Killing of ESKAPE cells by Hst 5**. *E. faecium* **(A)**, *S. aureus* **(B)**, *K. pneumoniae* **(C)**, *A. baumannii* **(D)**, *P. aeruginosa* **(E)**, and *E. cloacae* **(F)** cells in exponential growth were exposed to 30 μM Hst 5 in 10 mM NaPB or 100 mM NaPB for 1 min, 1, and 5 h. Aliquots taken at different time points were diluted and plated. CFU were determined after 24 h. Error bars represent the standard errors from at least three independent replicates of each strain.

In order to determine the bactericidal activity of Hst 5 against ESKAPE pathogens, six clinical isolates (Table 1) were tested using bactericidal assays (performed in 10 and 100 mM NaPB buffer) to determine percent bacterial killing following incubation for 1 min, 1, and 5 h with 30 μM Hst 5. *E. faecium* showed time-dependent killing since only 18.1 ± 8.9 percent of cells were killed by Hst 5 at 1 h, while killing was enhanced to 63.5 ± 3.5 percent after 5 h incubation (Figure 1A). *S. aureus* cells incubated for only 1 min with 30 μM Hst 5 showed 28.3 ± 2.3 percent killing which increased to 60.3 ± 5.7 and 69.7 ± 0.2 percent after 1 and 5 h, respectively (Figure 1B). A MRSA strain of *S. aureus* had lower but still significant killing by Hst 5 after 1 h (33.8 ± 3.5%). Interestingly, Hst 5 did not show any killing activity against *K. pneumoniae* for up to 5 h of incubation (Figure 1C). *A. baumannii* AB307-0294 had substantial sensitivity to Hst 5 with 31.4 ± 3.7, 90.0 ± 1.1, and 95. ± 2.3 percent killing at 1 min, 1, and 5 h, respectively (Figure 1D), while the *A. baumannii HUMC1* strain had even higher sensitivity with 100% killing at 1 h. Similarly, Hst 5 also showed strong killing activity against *P. aeruginosa* 94-323-0635, with a faster rate of killing. At just 1 min of incubation, 83.2 ± 1.3 percent killing was observed; while over 99.9 percent of cells were killed after 1 h treatment (Figure 1E). *P. aeruginosa* PAO1 had identical sensitivity to Hst 5 with 100% of cells killed at 1 h. *E. cloacae* sensitivity was similar to that of *A. baumannii* showing 20.3 ± 1.5, 66.1 ± 0.6, and 78.1 ± 1.7 percent of cells killed by Hst 5 at 1 min, 1 h, and 5 h, respectively (Figure 1F).

Since the activity of Hst 5 against *C. albicans* is abolished at higher phosphate buffer concentrations (Helmerhorst et al., 1999), Hst 5 killing activity against ESKAPE strains was also tested in a buffer with higher ionic strength. Killing activities of Hst 5 against *E. faecium, P. aeruginosa*, and *E. cloacae* were greatly reduced (Figures 1A,E,F) when carried out in 100 mM NaPB. However, bactericidal activity against *S. aureus* at 100 mM NaPB was similar to that observed in 10 mM NaPB (Figure 1B), while killing efficiency for *A. baumannii* were decreased by about 30 percent in 100 mM NaPB after 1 or 5 h incubation (Figure 1D). Effects of PBS on killing were similar to those seen in 100 mM NaPB (data not shown).

Based on these bactericidal results, we next tested growth inhibition by Hst 5 for these ESKAPE pathogens by determining MIC values in 10% MH broth (since Hst 5 is inactive in undiluted broth). Hst 5 MIC values of 38, 47, and 90 μM were observed for *A. baumannii, P. aeruginosa*, and *E. cloacae*; while we could not determine MIC values for *E. faecium* and *S. aureus* due to media components that inactivate Hst 5 (Table 2).

**Table 2:** **Hst 5-mediated killing of ESKAPE pathogens**.

	**% Hst 5 killing^a^**	**% Hst 5 biofilmKilling^b^**	**% Hst 5-spd killing^c^**	**Main mechanism of killing**		**MIC (μM)^d^**
				**Membrane disruption**	**Energy dependent**	
*Enterococcus faecium*	63.6	ND	96.6	-	+	ND
*Staphylococcus aureus*	69.8	3.2	69.2	-	-	ND
*Klebsiella pneumoniae*	0.0	19.5	0.0	N/A	N/A	N/A
*Acinetobacter baumannii*	95.34	15.1	96.8	+	-	38
*Pseudomonas aeruginosa*	>99.9	59.5	>99.9	+	-	47
*Enterobacter cloacae*	78.1	ND	90.1	-	+	90

a *Hst 5 (30 μM) killing efficiency of planktonic cells in 10 mM NaPB buffer for 5 h*.

b *Hst 5 (30 μM) killing efficiency of biofilm cells in 10 mM NaPB buffer for 1 h*.

c *Hst 5-Spd (30 μM) killing efficiency in 10 mM NaPB buffer for 5 h*.

d *MIC in 10% MH broth*.

*ND, Not Determined; N/A, Not Applicable*.

The activity of Hst 5 against ESKAPE pathogens grown as biofilms was determined after 16 h growth of each bacteria in 96-well plates (Table 2). Biofilms were treated with 30 μM Hst 5 for 1 h, dead cells stained with PI, and percent killing calculated. Neither *E. faecium* or *E. cloacae* formed robust biofilms in our hands so that Hst 5 killing could not be reliably determined for these two pathogens. As for planktonic cells, both strains of *P. aeruginosa* in biofilms were effectively killed by Hst 5 after 1 h (59.5%); however Hst 5 killing of *A. baumannii* biofilm cells was reduced to 15.1%; while killing of *S. aureus* biofilm cells (both strains) was negligible (5.1%). Surprisingly, we observed that Hst 5 killed 19.5% of *K. pneumoniae* when in biofilms, although it had no activity against planktonic cells.

Overall, our results show that Hst 5 has a strong bactericidal activity against *P. aeruginosa* and *E. cloacae* (and weaker killing with *E. faecium*) at lower ionic strength environments; while exerting killing against *A. baumannii* and *S. aureus* over a range of ionic strengths. However, biofilm cells of *A. baumannii* and *S. aureus* were more resistant to Hst 5 killing. To confirm these findings in real time and to assess whether ionic strength may influence metabolic or membrane lytic mechanisms of killing, we next measured Hst 5 binding, uptake, and time of killing for these ESKAPE pathogens in 10 mM NaPB.

### Bactericidal activity of Hst 5 against *E. faecium* and *E. cloacae* requires internalization and is energy dependent

**Figure 2:**
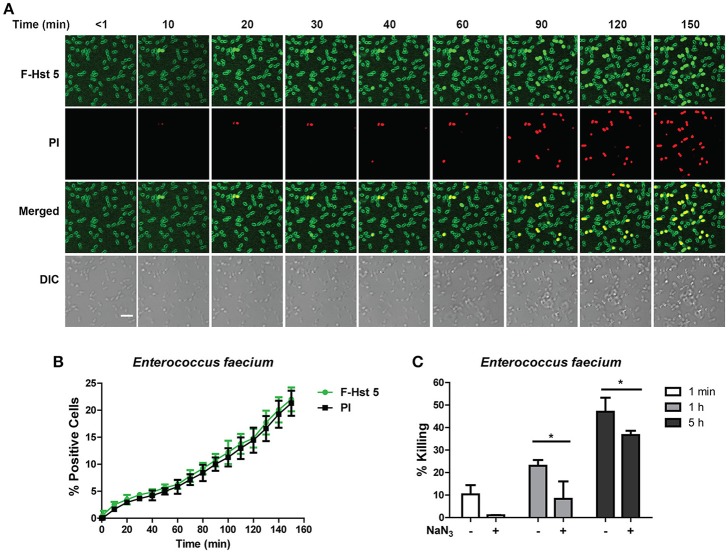
**Antibacterial activity of Hst 5 against ***E. faecium*** required internalization and is energy dependent. (A)** *E. faecium* cells were exposed to F-Hst 5 (30 μM) and PI (2 μg/mL). F-Hst 5 (green) and PI (red) uptake were measured in parallel by time-lapse confocal microscopy. Images were recorded every 10 min and selected images of indicated time points were shown. (Scale bar: 5 μm) **(B)** Quantitative analysis of F-Hst 5 uptake (green line) and PI uptake. Error bars represent the standard errors from four different fields of image. **(C)** Cells pretreated with 10 mM NaN_3_ at 37°C for 3 h showed decreased susceptibility to Hst 5. (^*^P < 0.05, Student's *t*-test).

Time-lapse confocal microscopy showed that Hst 5 (30 μM) rapidly (< 1 min) bound with the surface of *E. faecium* cells, although no internalization was evident until after 10 min of incubation (Figure 2A). This slow internalization resulted in only 15% of cells taking up F-Hst 5 by 120 min; but in each instance, Hst 5 internalization was accompanied by PI uptake (indicative of cell death; Figure 2B). This close correlation between intracellular Hst 5 and PI uptake suggested that targets of Hst 5 are intracellular rather than its effects being membrane lytic. However, the percentage of killing from the bactericidal assay was higher than apparent PI staining, suggesting that some portion of killing was delayed. To evaluate this, we pretreated cells with an inhibitor of energy metabolism (NaN_3_) as a means to determine whether the killing activity of Hst 5-treated *E. faecium* might be dependent upon cell metabolism. As expected, pretreatment of cells with NaN_3_ significantly reduced the killing efficiency of Hst 5 at 1 and 5 h (Figure 2C), showing that some portion of Hst 5 killing of *E. faecium* requires target cell energy, likely for Hst 5 internalization or intracellular organelle localization.

**Figure 3:**
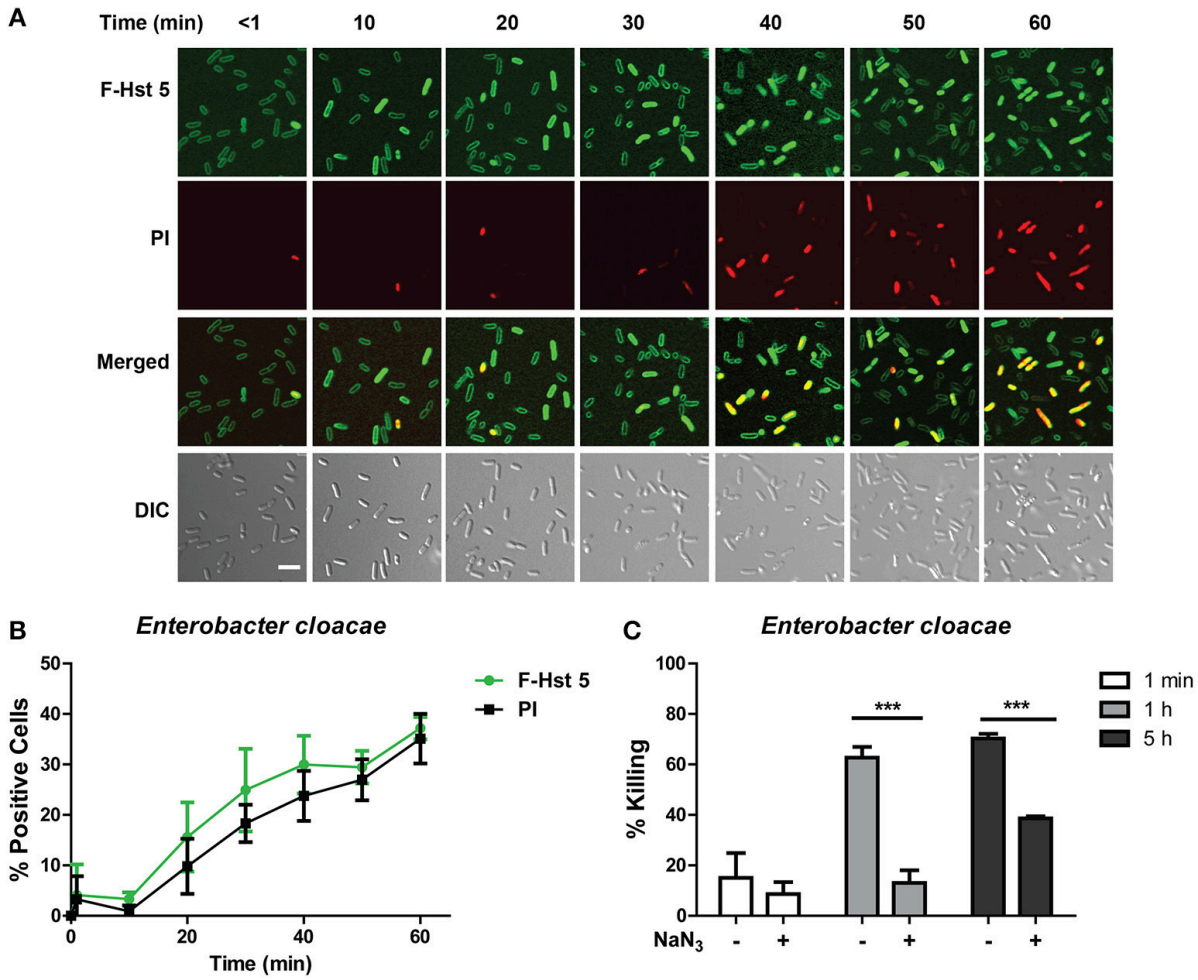
**Antibacterial activity of Hst 5 against ***E. cloacae*** requires internalization and is energy dependent. (A)** *E. cloacae* cells were exposed to F-Hst 5 (30 μM) and PI (2 μg/mL). The F-Hst 5 (green) and PI (red) uptake were measured in parallel by time-lapse confocal microscopy. Images were recorded every 10 min and selected images as indicated time points were shown. (Scale bar: 5 μm) **(B)** Quantitative analysis of F-Hst 5 uptake (green line) and PI uptake. Error bars represent the standard errors from four different fields of image. **(C)** Cells pretreated with 10 mM NaN_3_ at 37°C for 3 h showed more resistance to Hst 5. (^***^P < 0.001, Student's *t*-test).

*E. cloacae* cells had a very similar response when treated with F-Hst 5, in that the peptide associated with the surface of all cells almost immediately, however entry of F-Hst 5 (and accompanying PI staining) was slow and only occurred in 20% of cells within 30 min (Figure 3A). As for *E. faecium*, there was a close correlation between intracellular Hst 5 and PI uptake at all time points (Figure 3B), also pointing toward intracellular targets for bactericidal activity. *E. cloacae* cells pretreated with 10 mM NaN_3_ prior to exposure with 30μM Hst 5 were more resistant to the killing action of F-Hst 5 (Figure 3C), similar to the energy dependent mechanism found with *E. faecium*.

### Membrane disruption is involved in bactericidal activity of Hst 5 against *P. aeruginosa* and *A. baumannii*

**Figure 4:**
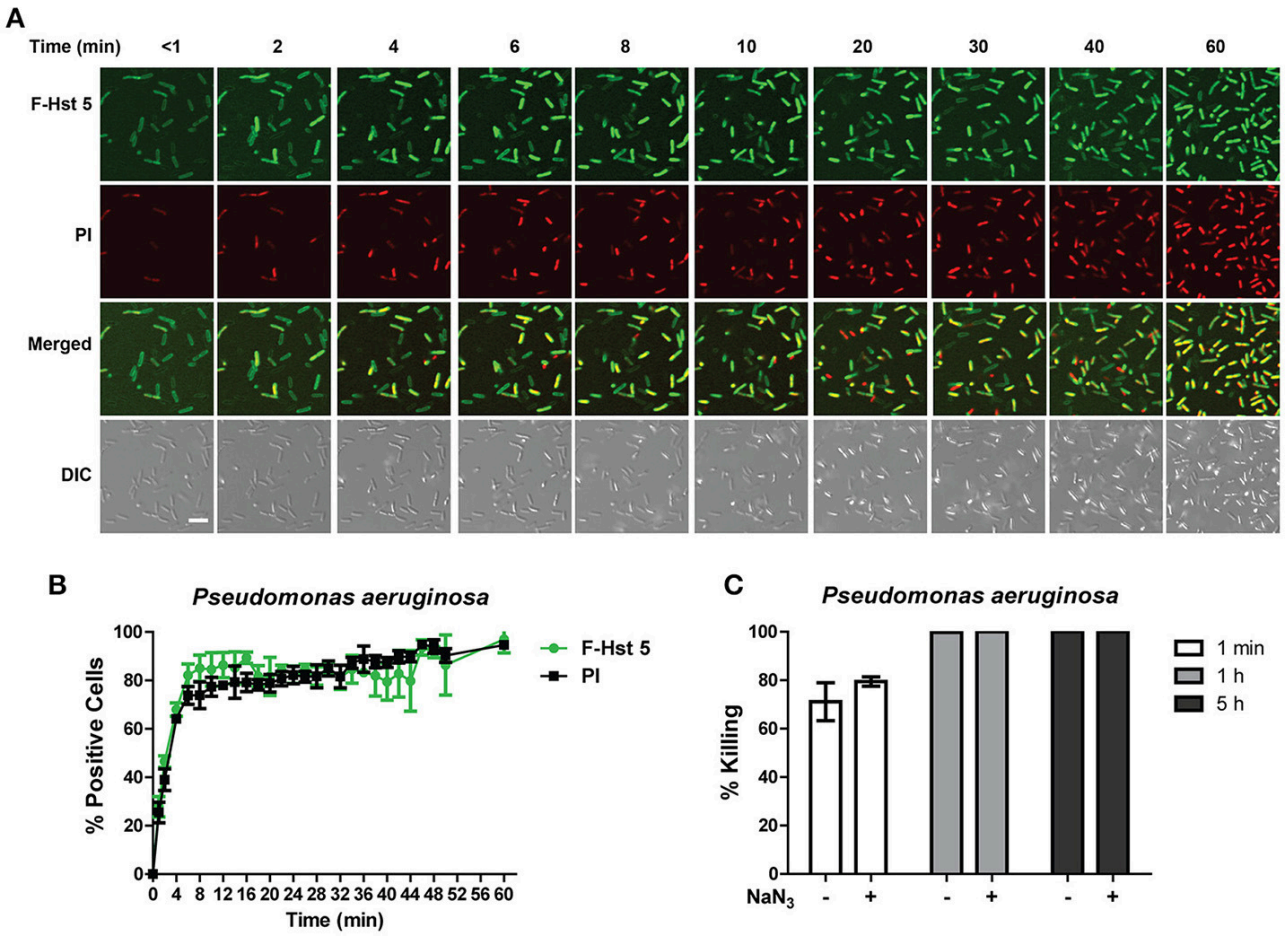
**Hst 5 bactericidal activity against ***P. aeruginosa*** cells is primarily mediated by membrane disruption. (A)** *P. aeruginosa* cells were exposed to F-Hst 5 (30 μM) and PI (2 μg/mL). F-Hst 5 (green) and PI (red) uptake were measured in parallel by time-lapse confocal microscopy. Images were recorded every 10 min and selected images at indicated time points were shown (Scale bar: 5 μm) **(B)** Quantitative analysis of F-Hst 5 uptake (green line) and PI uptake. Error bars represent the standard errors from four different fields of image. **(C)** Cells pretreated with 10 mM NaN_3_ at 37°C for 3 h did not show significant difference in susceptibility to Hst 5.

In agreement with its high bactericidal activity, F-Hst 5 completely covered the cell surface of *P. aeruginosa* within 1 min, and PI positive cells could be visualized within 2 min (Figure 4A). Quantitative analysis showed that 75% of cells contained F-Hst 5 and were PI positive within 5 min of addition of F-Hst 5 (Figure 4B). Both intracellular localization of Hst 5 and PI staining were rapid and simultaneous, pointing toward membrane lytic activity of Hst 5 mediated killing of *P. aeruginosa*. Pretreatment of *P. aeruginosa* cells with 10 mM NaN_3_ did not reduce the killing activity of Hst 5 (Figure 4C), also suggesting energy-independent membrane disruption as the main pathway for Hst 5 killing activity against *P. aeruginosa*.

**Figure 5:**
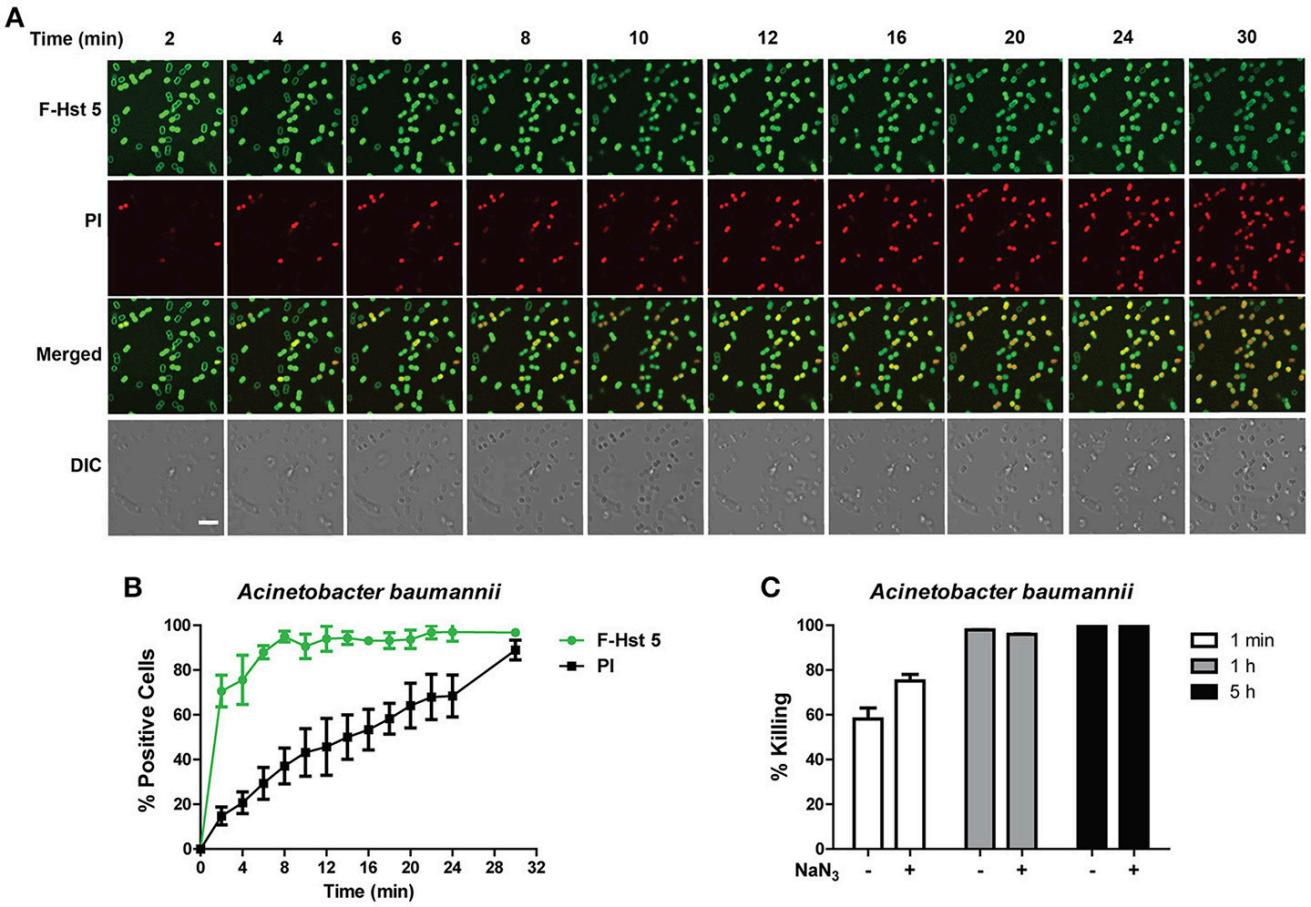
**Activity of Hst 5 against ***A. baumannii*** is mediated in part by membrane disruption. (A)** *A. baumannii* cells in exponential phase were exposed to F-Hst 5 (30 μM) and PI (2 μg/mL). The F-Hst 5 (green) and PI (red) uptake were measured in parallel by time-lapse confocal microscopy. Images were recorded every 2 min and selected images as indicated time points were shown. (Arrow, cells of F-Hst 5 uptake positive but without PI uptake; Scale bar: 5 μm). **(B)** Quantitative analysis of F-Hst 5 uptake (green line) and PI uptake. Error bars represent the standard errors from four different fields of image. **(C)** Cells pretreated with 10 mM NaN_3_ at 37°C for 3 h did not show significant difference in susceptibility to Hst 5.

*A. baumannii* showed a very similar profile to *P. aeruginosa* after exposure to F-Hst 5 in that F-Hst 5 was associated with the surface of all cells within 2 min; and PI positive cells were visualized with 2 min (Figure 5A). Although intracellular uptake of F-Hst 5 was very rapid with 70% of cells containing F-Hst 5 in just 2 min, PI staining did not occur simultaneously so that 70% PI positive cells was seen only after 25 min (Figure 5B). Interestingly, the portion of *A. baumannii* cells that showed slower PI staining already contained F-Hst 5; so we questioned whether bactericidal activity of these cells was energy-dependent. However, NaN_3_-pretreated *A. baumanni* cells had no difference in F-Hst 5 killing (Figure 5C), indicating that Hst 5-mediated killing of *A. baumanni* is partially due to membrane lysis and is energy independent, although some portion of killing might also be a result of its effect on intracellular targets.

### Hst 5-mediated killing of *S. aureus* is delayed and energy independent

**Figure 6:**
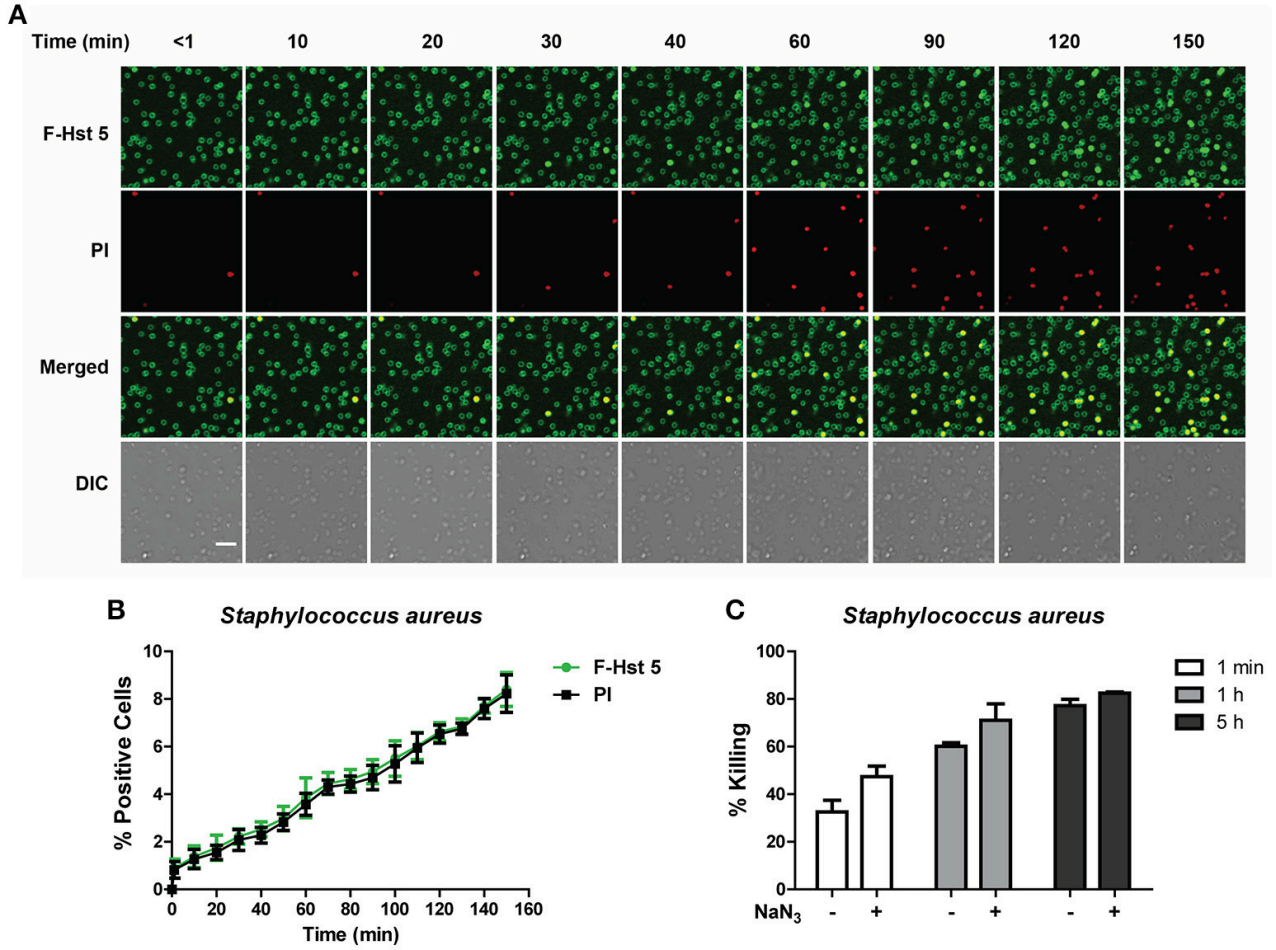
**The activity of Hst 5 against ***S. aureus*** is mediated by energy-independent mechanisms. (A)** *S. aureus* cells were exposed to F-Hst 5 (30 μM) and PI (2 μg/mL). F-Hst 5 (green) and PI (red) uptake were measured in parallel by time-lapse confocal microscopy. Images were recorded every 10 min and selected images of indicated time points were shown. (Scale bar: 5 μm). **(B)** Quantitative analysis of F-Hst 5 uptake (green line) and PI uptake. Error bars represent the standard errors from four different fields of image. **(C)** Cells pretreated with 10 mM NaN_3_ at 37°C for 3 h did not show significant difference in susceptibility to Hst 5.

F-Hst 5 was visualized binding to the cell surface of all *S. aureus* cells within 1 min after addition, however intracellular localization and PI uptake occurred slowly (Figure 6A), so that by 120 min only 7% of cells contained F-Hst 5 and were PI positive (Figure 6B). This was surprising since bactericidal activity of Hst 5 after 1 h incubation was found to have 60% killing (Figure 1). Furthermore, bactericidal assay with *S. aureus* pretreated with 10 mM NaN_3_ did not decrease the killing efficiency (Figure 6C). These results, combined with the salt-insensitive killing, suggest that there may be multiple targets for Hst 5-mediated killing of *S. aureus* that are non-lytic and energy independent.

### Hst 5 is ineffective in killing *K. pneumoniae* due to lack of sustained binding

**Figure 7:**
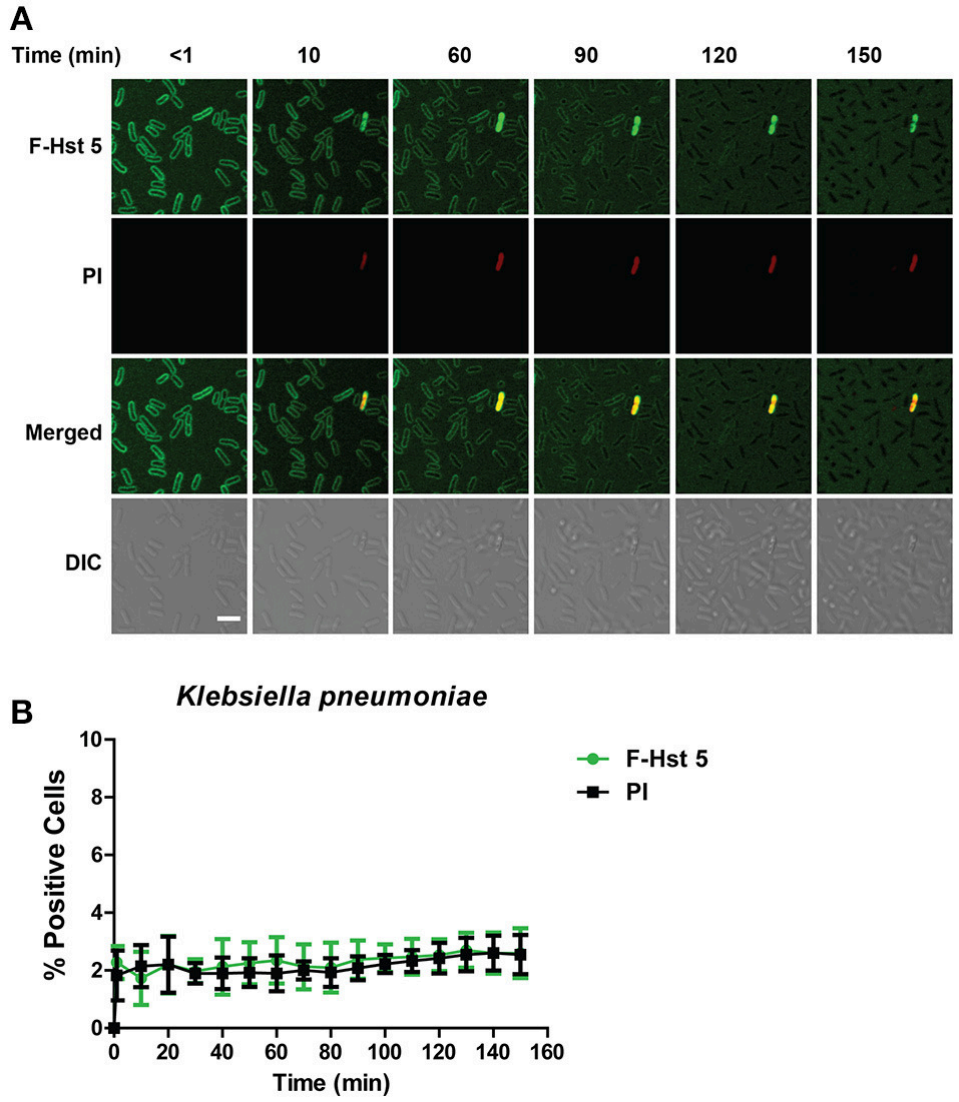
**Hst 5 is minimally active against ***K. pneumoniae***. (A)** *K. pneumoniae* cells in exponential phase were exposed to F-Hst 5 (30 μM) and PI (2 μg/mL). F-Hst 5 (green) and PI (red) uptake were measured in parallel by time-lapse confocal microscopy. Images were recorded every 10 min and selected images of indicated time points were shown. (Scale bar: 5 μm). **(B)** Quantitative analysis of F-Hst 5 uptake (green line) and PI uptake. Error bars represent the standard errors from four different fields of image.

Since Hst 5 showed negligible killing against *K. pneumoniae* (Figure 1C), we examined the reason for this using confocal microscopy. F-Hst bound to most *K. pneumoniae* cells within 2 min, however by 10 min most binding was lost suggesting detachment of Hst 5 from the surface capsule (Figure 7A). As expected, F-Hst 5 and PI uptake were extremely limited (only 2% of cells), even up to 150 min (Figure 7B). These results suggest that F-Hst 5 is released from the *K. pneumoniae* after initial binding (perhaps due to lower binding efficacy in the presence of its capsule) and thus Hst 5 is unable to gain entry or lyse the cells and therefore is ineffective against *K. pneumoniae*.

### Hst 5-Spd has improved bactericidal efficiency against ESKAPE pathogens that take up Hst 5

**Figure 8:**
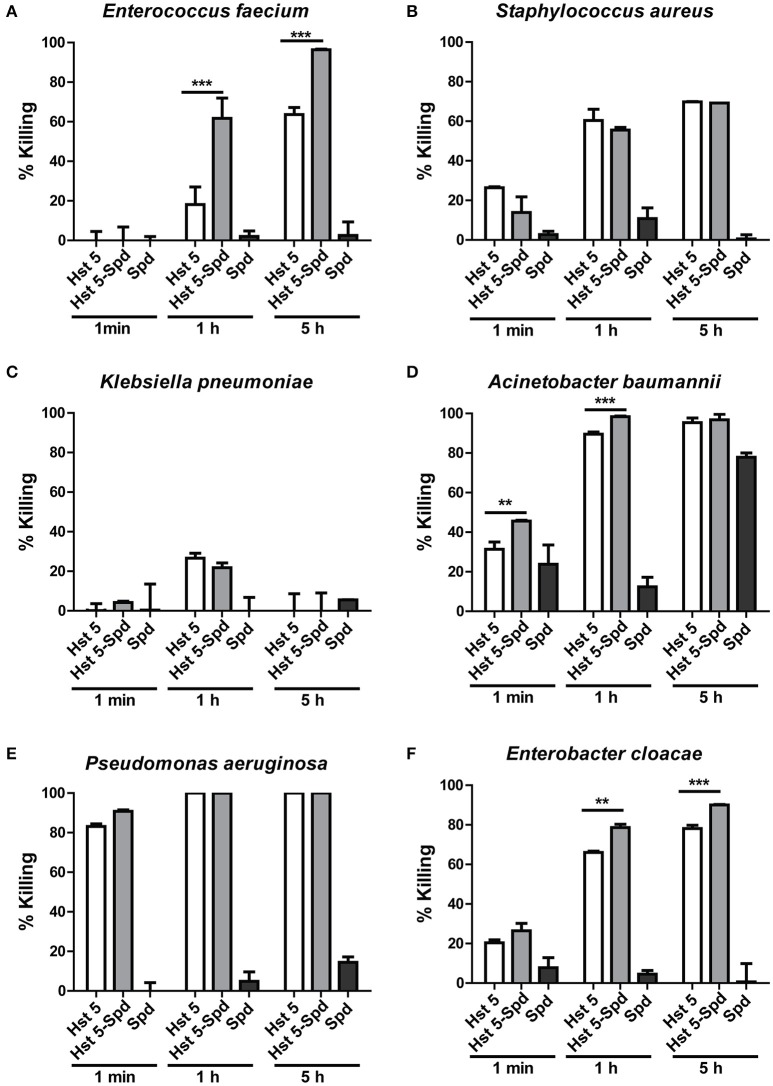
**Killing of ESKAPE pathogens by Hst 5-Spd**. *E. faecium* **(A)**, *S. aureus* **(B)**, *K. pneumoniae* **(C)**, *A. baumannii* **(D)**, *P. aeruginosa* **(E)**, and *E. cloacae* **(F)** cells in exponential growth were exposed to 30 μM of Hst 5, F-Hst 5 and spermidine in 10 mM NaPB for 1 min, 1, and 5 h. Aliquots taken at different time points were diluted and plated. CFU were determined after 24 h. Error bars represent the standard errors from at least three independent replicates of each strain. Hst 5-Spd conjugate showed more killing efficiency against *E. faecium* **(A)**, *A. baumannii* **(D)**, and *E. cloacae* **(F)** (^**^P < 0.01, ^***^P < 0.001, Student's *t*-test).

We have previously reported that spermidine-conjugated Hst 5 (Hst 5-Spd) has greater activity against *C. albicans* because the spermidine conjugate translocates more efficiently into the yeast cells (Tati et al., 2014). Therefore, to determine if Hst 5-Spd has improved bactericidal activity against ESKAPE pathogens as well, we tested the killing activity of Hst 5-Spd [30 μM] against all the six strains (Figure 8). Interestingly, Hst 5-Spd had significantly higher killing activity compared with Hst 5 against *E. faecium, E. cloacae*, and *A. baumannii* (Figure 8). Hst 5-Spd bactericidal effects were most improved against *E. faecium*; increased by three-fold after 1 h incubation and by 1.5-fold after 5 h, while Spermidine (Spd) alone had no killing activity even after 5 h treatment (Figure 8A). Hst 5-Spd bactericidal activity was also increased significantly in *E. cloacae* compared with Hst 5, although by only 15% at 1 h and at 5 h. Hst 5-Spd also showed a small but significant increase in killing activity against *A. baumannii* compared with Hst 5 (23% at 1 min and 10% at 1 h). However, after 5 h there was no significant difference perhaps due to the high killing that occurred as a result of Spd (30 μM) itself (Figure 8D). These three species of ESKAPE pathogens were also ones that we found to involve Hst 5 uptake and intracellular targets (Figures 2, 3, 5), suggesting that improved Hst 5-Spd activity in these strains is a result of improved uptake. This is supported by our data showing that the killing ability of Hst 5-Spd against *P. aeruginosa* was not improved, in agreement with a membrane lytic mechanism for this organism. Furthermore, the Hst 5 resistant pathogen *K. pneumoniae* was not affected by Hst 5-Spd showing that this conjugate does not improve binding to this encapsulated ESKAPE pathogen.

## Discussion

Here we report the remarkable finding that although Hst 5 activity has been believed to be limited to fungi, Hst 5 also demonstrates very high bactericidal activity against several ESKAPE pathogens. Up to now, a few reports have described interactions between Hst 5 and bacteria. Hst 5 was found to have low killing against *Streptococcus gordonii* (a Gram-positive commensal bacterium of the human oral cavity; Andrian et al., 2012). Zinc-mediated killing of *E. faecalis* bacteria by histidine–rich histatin analogs was found (Rydengard et al., 2006), and killing activity of another histatin derivative (P113) against *P. aeruginosa* and *S. aureus in vitro* was shown (Giacometti et al., 2005). Hst 5 may have other non-bactericidal activities in that it attenuates chemokine responses by binding to *Porphyromonas gingivalis* hemagglutinin B (Borgwardt et al., 2014). Here we expand the scope of Hst 5 activity by showing that full length Hst 5 and an Hst 5 spermidine conjugate both exert very significant killing of all but one ESKAPE pathogen by multiple mechanisms.

The mechanisms by which Hst 5, a naturally occurring salivary protein, kills *C. albicans* have been well-studied and suggest the involvement of multiple intracellular targets (Puri and Edgerton, 2014). The effects on fungal targets range from non-lytic leakage of ATP and K^+^ ions to mitochondrial damage and oxidative stress generation (Puri and Edgerton, 2014); and more recently, potential metal scavenging causing nutritional stress (Puri et al., 2015). However, it has been clearly shown that the killing is non-lytic and energy dependent. Interestingly, the ambiguity in the mechanisms of antimicrobial peptides seems universal. Many antibacterial peptides that were considered to be purely lytic are now believed to involve other killing mechanisms ranging from bacterial cell wall disruption to effects on protein and nucleic acid synthesis (Brogden, 2005). We found that Hst 5 can efficiently kill ESKAPE pathogens killing using a unique blend of lysis and energy dependency.

Antibiotic resistance has been a challenge for treating drug resistant bacterial infections and ESKAPE pathogens are no exception (Rice, 2010). One classic drug resistance mechanism entails efflux of the antimicrobial molecules by bacterial cells (Sun et al., 2014). However, Hst 5 resistance is mediated by efflux through *C. albicans* Flu1 transporters (Li et al., 2013) and is not likely to occur in bacteria, as there are no similar transporters known in bacteria. In contrast, *C. albicans* Dur3 transporters (used in polyamine uptake in fungi) are needed for Hst 5 uptake and candidacidal activity (Kumar et al., 2011). Interestingly all ESKAPE pathogens have spermidine/polyamine transporter homologs (Palmer et al., 2010; Ren et al., 2010; Park et al., 2011; Liu et al., 2012; Yao and Lu, 2014; Wang et al., 2016) that might mediate Hst 5 uptake in pathogens that require internalization for killing. Also, the improved bactericidal activity of the Hst 5-spermidine conjugate against *E. faecium* (whose spermidine transporter has the highest homology to the *C. albicans* Dur3 transporter, our unpublished data), and to some extent against *E. cloacae* and *A. baumannii* may be a result of better uptake due to similarity of polyamine transporters expressed in these cells. Biofilm formation is another resistance mechanism that leads to poor drug penetration and accessibility (del Pozo and Patel, 2007; Hoiby et al., 2010). It has previously been shown that Hst 5 is effective against *C. albicans* biofilm (Konopka et al., 2010) and thus Hst 5 can potentially make its way through the polysaccharide extracellular biofilm matrix of ESKAPE bacteria as well.

Prophylactic antimicrobial therapy, especially when applied topically, can be of great advantage to prevent surgical, wound, and burn infections. However, depending on the specificity of the agent used, this may also lead to the killing of healthy flora at the site of application that prevents colonization of pathogens. Here we show that salivary Hst 5 that has limited antibacterial activity against most human oral commensal organisms including *Streptococcal* sp. (found on the human skin; Dale and Fredericks, 2005; Belkaid and Segre, 2014), is extremely effective against *P. aeruginosa* and *A. baumanni*. This provides a novel potential therapeutic application for Hst 5 since *P. aeruginosa* is one of the most important causative agents for burn infections (Tredget et al., 1992). Furthermore, *P. aeruginosa* is responsible for a majority of bacterial eye infections related to contact lens use (Cope et al., 2016). While Hst 5 is intrinsically absent from human tears, its exogenous application to treat such infections seems plausible, given the high killing activity of Hst 5 against *P. aeruginosa*. Potential therapeutic use of Hst 5 has some limitations that are inherent to its cationic nature. Since high salt conditions negatively affect Hst 5 microbicidal activity (Helmerhorst et al., 1999; Jang et al., 2010), this may restrict the use of Hst 5 for treatment of systemic disease, although the Hst 5 derivative P113 was found to be effective for systemic use in a rat model of *P. aeruginosa* sepsis (Cirioni et al., 2004). However Hst 5 has a great potential for application topically, both on human skin and in the eye, especially when carried in hypotonic gels and solutions.

Multiple drugs are sometimes required to completely eradicate drug resistant infections. Although further testing of additional strains of ESKAPE pathogens needs to be done, we show here the killing activity of Hst 5 against ESKAPE pathogens, taken together with how Hst 5 can potentially affects multiple intracellular targets, presents the possibility of using this protein in synergy with other existing antibiotics.

## Author contributions

Conceived and designed the experiments: ME, HD, SP. Performed the experiments: HD, AM, HN. Analyzed the data: HD, AM, HN. Prepared the paper: ME, HD, SP, TR.

## Funding

This work was supported by NIDCR grants DE10641 and DE022720 to ME.

### Conflict of interest statement

The authors declare that the research was conducted in the absence of any commercial or financial relationships that could be construed as a potential conflict of interest.
